# Effect of the blood cells, inflammatory cytokines, antibodies, circulating metabolome, and immune cells on skin cancers: A bidirectional 2-sample Mendelian randomization study and mediation analysis

**DOI:** 10.1097/MD.0000000000043233

**Published:** 2025-07-11

**Authors:** Jiahui Yao, Mingshuai Han

**Affiliations:** a Department of Emergency Surgery, Linping Campus, Second Affiliated Hospital, Zhejiang University School of Medicine, Hangzhou, Zhejiang Province, China.

**Keywords:** antibodies immune responses, blood cells, circulating metabolites, immune cells, inflammatory cytokines, Mendelian randomization, skin cancer

## Abstract

Previous research has highlighted the involvement of several human blood cells in skin cancer, but large-scale studies are lacking to explore their relationship and avoid confounding factors. Here, we comprehensively investigated the causal effect of blood cells on skin cancer subtypes across 4 different human microenvironments through 2-sample Mendelian randomization (MR) analysis and mediation analysis. Summary statistics of 91 human blood cells, 233 circulating metabolites, 731 immune cells, 46 antibody immune responses, 91 inflammatory cytokines, and 4 skin cancer traits (including cutaneous melanoma, nonmelanoma skin cancer, basal cell carcinoma, and squamous cell carcinoma) were derived from genome-wide association studies. The bidirectional 2-sample MR was used to determine the causality between exposures and outcomes. Additionally, comprehensive sensitivity analyses were performed to ensure the robustness of MR findings. Finally, the mediation analysis was applied to identify the role of blood cells in skin cancers mediated by 4 different microenvironments. MR revealed causal associations between 18 different types of human blood cells, 30 different types of circulating metabolites, 136 different types of immune cells, 17 different types of antibodies immune responses, 17 different types of inflammatory cytokines with skin cancers. Reverse MR analysis indicated skin cancers were causally associated with the levels of 4 different types of human blood cells. Mediation analysis revealed 19 mediation correlations during the causal effect from blood cells to skin cancers. Among them, 13 belonged to immune cells, 3 belonged to inflammatory cytokines, and 3 belonged to antibodies immune responses. Sensitivity analyses confirmed the consistency of these findings. This study represents the first comprehensive evaluation demonstrating causal relationships among human blood cells, circulating metabolites, immune cells, antibodies immune responses, inflammatory cytokines, and skin cancers, thereby providing novel insights and potential intervention targets for skin cancer treatment.

## 1. Introduction

Skin cancer is one of the most widespread cancers worldwide. According to its original classification, skin cancer can be classified into malignant melanoma (MM) and other malignant neoplasms of skin (OMNS). The OMNS can be later divided into squamous cell carcinoma (SCC) and basal cell carcinoma (BCC). According to the statistics of global cancer, OMNS constitutes approximately 95% of skin cancer cases, while MM only accounts for 2% of skin cancer cases.^[1]^ However, compared to the low mortality and high incidence rate of OMNS, MM is known to cause early metastasis and as high as an 80% mortality rate.^[2]^ For MM patients, early detection and effective therapy can significantly reduce their mortality rates; thus, identifying the risk factors for early prevention and intervention is urgently needed.^[3]^ Apart from surgical excision, immunotherapy and targeted therapy are also important for skin cancer treatment. To avoid tumor resistance, exploring innovative therapeutic targets is also needed.^[4]^

Although the specific causes of skin cancer remain unclear, multiple relevant studies have shown that several factors play a pivotal role in the progression, occurrence, and metastasis of skin cancer, including inflammatory factors,^[5]^ immune system,^[6]^ and metabolites.^[7]^ For example, lipid metabolites leukotrienes and prostaglandins, can promote the progression of skin cancer through modulating the inflammatory response.^[8,9]^ Macrophages and T cells are both core components of immune system. The differentiation of macrophages into M2 or generation of CD4 + T cells can significantly facilitate skin cancer development.^[10]^ Meanwhile, immune cells can influence tumor progression by regulating the tumor microenvironment.^[11,12]^ Exploring the mechanisms of these microenvironments can provide novel insights for the treatment of skin cancer patients.

Live human blood cells can reflect the environmental factors, states, and physiological processes of several diseases. For example, the progression of atherosclerosis and insulin resistance can result from the dysregulation of hematopoietic processes.^[13,14]^ Cells in peripheral blood may provide a diagnostic window for integrative physiology and multiple organ systems.^[15,16]^ Concerning its role in cancer, previous studies have reported that blood cells such as B cells and eosinophils are connected with the prognosis of skin cancers.^[17,18]^ However, previous studies mainly rely on observational and experimental analyses to explore the roles of human blood cells in skin cancers, whereas this method is causally influenced by confounding factors and reverse causation in observational studies.

Mendelian randomization (MR) is an effective epidemiological method for inferring etiology, enabling more accurate determination of correlations between skin cancer and risk factors. This method applies genetic variants as instrumental variables (IVs) to explore causal relationships. Compared with traditional observational studies, MR is less influenced by reverse causation and confounders.^[19,20]^ Although the close association between immune cells, circulating metabolites, inflammatory cytokines, and skin cancers by MR analysis, their mediation effects on blood cells to skin cancers remains unknown. Consequently, this study employs bidirectional MR analysis to investigate potential causal relationships between immune cells, circulating metabolites, inflammatory cytokines, blood cells, and skin cancers, aiming to enhance understanding of stroke pathogenesis and offer insights for its diagnosis and therapy.

## 2. Materials and methods

### 2.1. Study design

**Figure 1.**
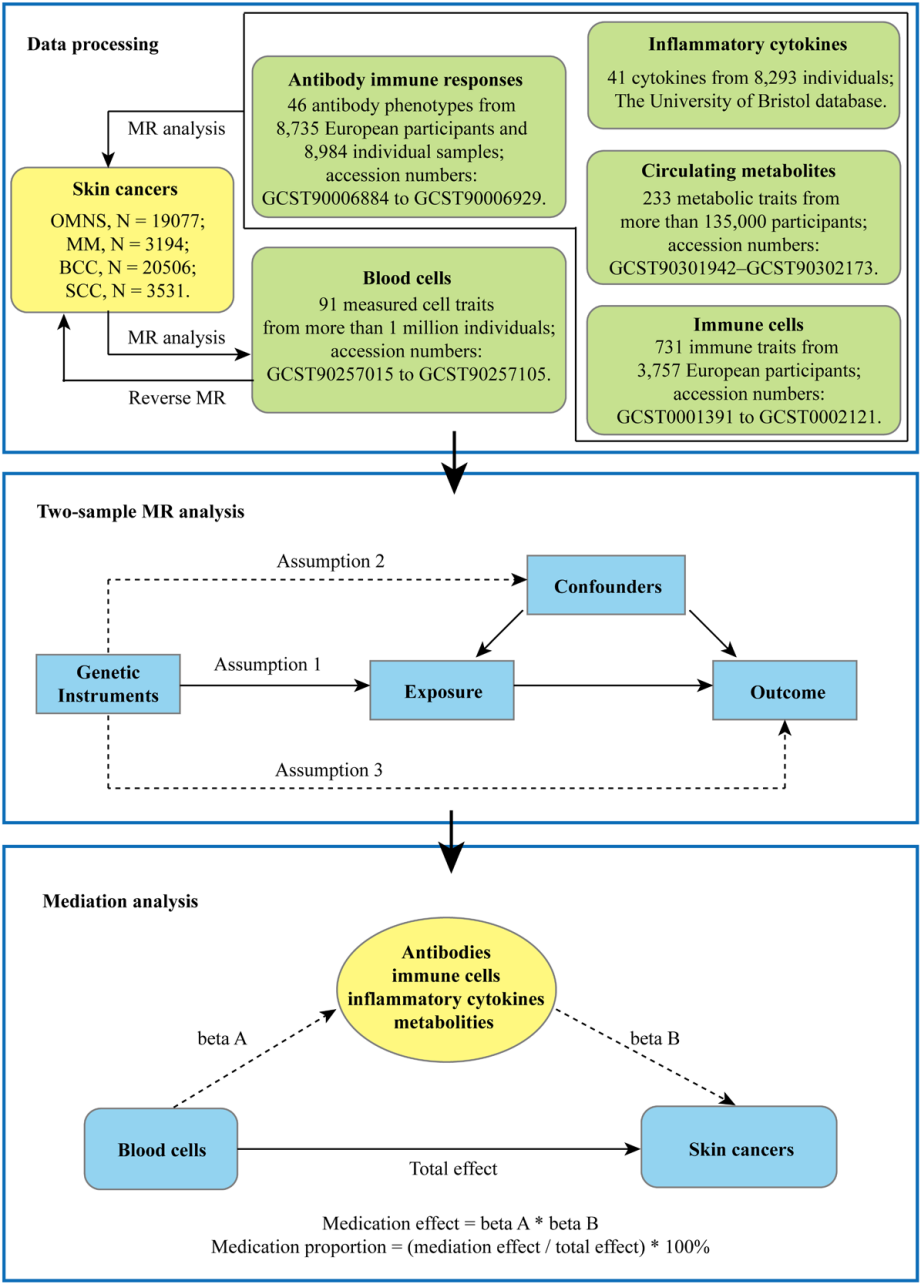
Schema of the study.

The general design of this study is depicted in Figure 1. It consists mainly of 2 steps. First, 2-sample bidirectional MR analyses were conducted to assess the causal relationship between human blood cells, circulating metabolites, immune cells, antibodies immune responses, inflammatory cytokines, and skin cancers. Second, multivariable MR analyses were used to investigate the mediating effect of circulating metabolites, immune cells, antibodies immune responses, and inflammatory cytokines on the association between blood cells and skin cancers. All summary statistics were extracted from the public genome-wide association study (GWAS) database; thus, ethical approval or participant consent was not required for this study. The MR analyses were conducted following the Strengthening the Reporting of Observational Studies in Epidemiology guidelines.^[21]^

### 2.2. GWAS data sources

Previous genome-wide association studies provided GWAS summary data of blood cells,^[22]^ circulating metabolites,^[23]^ immune cells,^[24]^ antibodies immune responses,^[25]^ and inflammatory cytokines,^[26]^ respectively. The human blood cells summary statistics were obtained from the GWAS Catalog (https://gwas.mrcieu.ac.uk) under accession numbers GCST90257015 to GCST90257105. These measured cell traits were obtained from >1 million individuals. In order to elicit phenotypes that are latent at baseline, blood cell properties are assessed not only on clinically available assays of cross-sectional cellular counts, but also under perturbation conditions. The GWAS data for 46 antibody immune response phenotypes were also obtained from the GWAS Catalog from GCST90006884 to GCST90006929, which include 8735 European individual participants and 8984 individual samples representing 13 pathogens. Summary statistics of circulating metabolomics were acquired from the accession numbers GCST90301942–GCST90302173, which included 233 metabolic traits from >135,000 participants. The University of Bristol publican database (https://data.bris.ac.uk/data/), which includes the Cardiovascular Risk in Young Finns Study, FINRISK2002, and FINRISK1997 3 cohort studies, was applied for collecting 41 inflammatory cytokines from 8293 individuals. A total of 731 immune traits were included from the GWAS accession numbers from GCST0001391 to GCST0002121. These data were performed from 3757 European participants.

In this study, skin cancers, obtained from the FinnGen biobank (https://www.finngen.fi/en, version: R10), were categorized into 4 subtypes: MM with 3194 patients and 314,193 controls (https://storage.googleapis.com/finngen-public-data-r10/summary_stats/finngen_R10_C3_MELANOMA_SKIN_EXALLC.gz); OMNS with 19,077 patients and 314,193 controls (https://storage.googleapis.com/finngen-public-data-r10/summary_stats/finngen_R10_C3_OTHER_SKIN_EXALLC.gz); SCC with 3531 patients and 314,193 controls (https://storage.googleapis.com/finngen-public-data-r10/summary_stats/finngen_R10_C3_SQUOMOUS_CELL_CARCINOMA_SKIN_EXALLC.gz); BCC with 20,506 patients and 314,193 controls (https://storage.googleapis.com/finngen-public-data-r10/summary_stats/finngen_R10_C3_BASAL_CELL_CARCINOMA_EXALLC.gz). The details of all skin cancer sets are summarized in Table 1.

**Table 1:** Details of the skin cancers included in the Mendelian randomization.

Trait	Sample size	Control size	Link
BCC	20,506	314,193	https://storage.googleapis.com/finngen-public-data-r10/summary_stats/finngen_R10_C3_BASAL_CELL_CARCINOMA_EXALLC.gz
OMNS	19,077	314,193	https://storage.googleapis.com/finngen-public-data-r10/summary_stats/finngen_R10_C3_OTHER_SKIN_EXALLC.gz
MM	3194	314,193	https://storage.googleapis.com/finngen-public-data-r10/summary_stats/finngen_R10_C3_MELANOMA_SKIN_EXALLC.gz
SCC	3531	314,193	https://storage.googleapis.com/finngen-public-data-r10/summary_stats/finngen_R10_C3_SQUOMOUS_CELL_CARCINOMA_SKIN_EXALLC.gz

BCC = basal cell carcinoma, MM = malignant melanoma, OMNS = other malignant neoplasms of skin, SCC = squamous cell carcinoma.

### 2.3. IVs selection

Effective bidirectional MR analysis has to meet 3 hypotheses: IVs are significantly connected with exposure; IVs serve as an independent factor of confounders; IVs only affect results through exposure factors. Assuming these 3 assumptions are met, we set the significance threshold of each IV to *P* < 5 × 10^−5^ to ensure a sufficient number of SNPs. Linkage disequilibrium was controlled by carrying out the clumping process with r2 < 0.001 and a window size = 10,000 kb. In addition, SNPs with pleiotropy effect (*P* > .05) were eliminated using the MR pleiotropy residual sum and outlier (MR-PRESSO) test, and all palindromic SNPs were also removed. In order to prevent instrument bias, the F-statistic for each IV was computed as β2/σ2 (β represents the beta coefficient for the SNP-exposure association, and σ denotes variance). Weak IVs with F-statistic < 10 were excluded in this study to avoid significant deviations. The same clumping parameter was also employed in the reverse analysis.

### 2.4. Statistical analysis

All MR analyses were conducted using the “TwoSampleMR” R package to estimate the correlation between antibody immune responses, cerebrospinal fluid metabolites, and stroke. Causality was inferred using several analytical methods, including inverse variance-weighted (IVW), weighted median, MR-Egger, simple mode, and weighted mode methods. The IVW model was the primary approach in this study. Additionally, sensitivity analyses, such as the MR-PRESSO test, MR-Egger intercept test, and leave-one-out analysis, were performed to assess the robustness of identified causal relationships. Horizontal pleiotropy was assessed using the MR-PRESSO global test and MR-Egger intercept analysis, while heterogeneity was assessed using Cochrane Q statistic test. A significance threshold of *P* < .05 was set for horizontal and heterogeneity analyses. Leave-one-out analysis was conducted to examine the influence of specific SNPs on significant results.

Additionally, reverse MR analyses were also performed to explore the causal role of skin cancers on blood cells, aiming to assess feedback loops between diseases and risk factors. In this context, skin cancer subtypes were considered as exposures, and blood cells were treated as outcomes. The analysis process was akin to that of forward MR analysis.

Mediation analysis was applied to evaluate the potential mediation effect of circulating metabolites, immune cells, antibodies immune responses, and inflammatory cytokines in the pathway from blood cells to skin cancers. The mediation effect was calculated as beta A * beta B (where beta A represents the causal effect of human blood cells on mediating factors, and beta B denotes the causal effect of mediating factors on skin cancers). The direct effect was derived from the total effect minus the mediation effect (where the total effect indicates the impact of blood cells on skin cancers).

## 3. Results

### 3.1. The causality between blood cells and skin cancers

**Figure 2.**
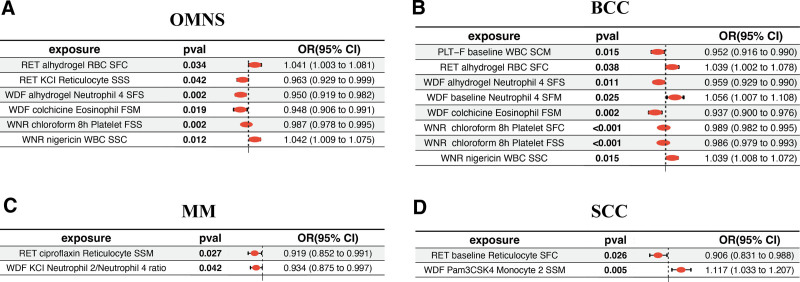
IVW analysis showed the causal associations between blood cells and skin cancers (A) OMNS, (B) BCC, (C) MM, and SCC. BCC = basal cell carcinoma, IVW = inverse variance-weighted, MM = malignant melanoma, OMNS = other malignant neoplasms of skin, SCC = squamous cell carcinoma.

After excluding blood cells with inconsistent odds ratio (OR) directions across the 5 MR methods and those showing pleiotropy (*P* > .05), we identified 18 association pairs between blood cells and skin cancers (see Table S1, Supplemental Digital Content, https://links.lww.com/MD/P431, which illustrates the causal impact of human blood cells on skin cancers). Figure 2A–D illustrates their causal relationships analyzed through IVW analysis. Notably, reticulocyte variability in side scatter standard deviation under KCl perturbation measured by reticulocyte dye (RET; RET-KCl-RET-side scatter standard) (OR = 0.963, 95% CI = 0.929–0.999), neutrophil 4 variability in side fluorescence standard (SFS) deviation under alhydrogel perturbation measured by WDF dye (WDF-Alhydrogel-NE-SFS) (OR = 0.950, 95% CI = 0.919–0.982), eosinophil variability in forward scatter median (FSM) under colchicine perturbation measured by WDF dye (WDF-Colchicine-EOS-FSM) (OR = 0.948, 95% CI = 0.906–0.991), platelet variability in forward scatter standard (FSS) deviation under 8h chloroform perturbation measured by WNR dye (WNR-chloroform 8 h-PLA-FSS) (OR = 0.987, 95% CI = 0.978–0.995) were negatively correlated with the risk of OMNS, whereas red blood cell variability in side fluorescence coefficient (SFC) under alhydrogel perturbation measured by RET dye (RET-Alhydrogel-RBC-SFC) (OR = 1.041, 95% CI = 1.003–1.081) and white blood cell variability in side scatter coefficient (SSC) under nigericin perturbation measured by WNR dye (WNR-Nigericin-WBC-SSC) were positively correlated with the risk of OMNS. Among MM patients, reticulocyte variability in side scatter median (SSM) under ciproflaxin perturbation measured by reticulocyte dye (RET-Ciproflaxin-RET-SSM) (OR = 0.919, 95% CI = 0.852–0.991) and neutrophil 2/neutrophil 4 ratio under KCl perturbation measured by WDF dye (WDF-KCl-NE2/NE4 ratio) (OR = 0.934, 95% CI = 0.875–0.997) were negatively correlated with their risk. For BCC patients, white blood cell variability in SSM at baseline measured by platelet-F dye (PLA-F-Baseline-WBC-SSM) (OR = 0.952, 95% CI = 0.916–0.990), neutrophil 4 variability in SFS deviation under alhydrogel perturbation measured by WDF dye (WDF-Alhydrogel-NE4-SFS) (OR = 0.959, 95% CI = 0.929–0.990), eosinophil variability in FSM under colchicine perturbation measured by WDF dye (WDF-Colchicine-EOS-FSM) (OR = 0.938, 95% CI = 0.900–0.976), and platelet variability in SFC or FSS under 8h chloroform perturbation measured by WNR dye (WNR-Chloroform 8h-PLA-SFC and WNR-Chloroform 8 h-PLA-FSS) (OR = 0.989, 95% CI = 0.982–0.995 and OR = 0.986, 95% CI = 0.979–0.993) were negatively associated with their risk, while red blood cell variability in SFC under alhydrogel perturbation measured by reticulocyte dye (RET-Alhydrogel-RBC-SFC) (OR = 1.039, 95% CI = 1.002–1.078), neutrophil 4 variability in side fluorescence median (SFM) at baseline measured by WDF dye (WDF-Baseline-NE4-SFM) (OR = 1.056, 95% CI = 1.007–1.108), and white blood cell variability in SSC under nigericin perturbation measured by WNR dye (WNR-Nigericin-WBC-SSC) (OR = 1.039, 95% CI = 1.008–1.072) levels showed a positive association. For SCC patients, reticulocyte variability in SFC at baseline measured by reticulocyte dye (RET-Baseline-RET-SFC) (OR = 0.906, 95% CI = 0.831–0.988) was negatively correlated with their risk, while monocyte 2 variability in SSM under Pam3CSK4 perturbation measured by WDF dye (WDF-Pam3CSK4-MON2-SSM) (OR = 1.117, 95% CI = 1.033–1.207) was positively correlated. There was no significant heterogeneity or horizontal pleiotropy among these causal relationships (see Table S2, Supplemental Digital Content, https://links.lww.com/MD/P431, which illustrates heterogeneity and pleiotropy analyses of human blood cells on skin cancers).

**Figure 3.**
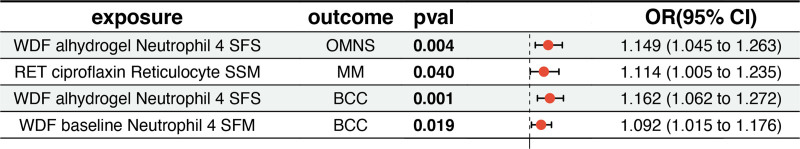
Reverse MR analysis showed the causal role of skin cancers on blood cells. MR = Mendelian randomization.

Furthermore, reverse MR analyses revealed that OMNS and MM had a significant positive impact on the levels of WDF-Alhydrogel-NE4-SFS (OR = 1.149, 95% CI = 1.045–1.263) and RET-Ciproflaxin-RET-SSM (OR = 1.114, 95% CI = 1.005–1.235), respectively. Meanwhile, BCC was significantly associated with WDF-Baseline-NE4-SFM (OR = 1.162, 95% CI = 1.062–1.271) and WDF-Alhydrogel-NE4-SFS (OR = 1.092, 95% CI = 1.015–1.176) (Fig. 3; see Table S3, Supplemental Digital Content, https://links.lww.com/MD/P431, which illustrates the causal impact of skin cancers on blood cells), thereby confirming the bidirectional relationship between human blood cells and skin cancers. No significant heterogeneity or horizontal pleiotropy was observed in reverse MR analysis (see Table S4, Supplemental Digital Content, https://links.lww.com/MD/P431, which illustrates heterogeneity and pleiotropy analyses of skin cancers on blood cells).

### 3.2. The causal effect of immune cells, inflammatory cytokines, circulating metabolites, and antibodies immune responses on skin cancers

Based on the IVW analysis (see Table S5, Supplemental Digital Content, https://links.lww.com/MD/P431, which illustrates the causal impact of immune cells, inflammatory cytokines, circulating metabolites, and antibodies immune responses on skin cancers), the results showed 136 causal associations between immune cells and skin cancer (P_IVW_ < 0.05, corresponding to 23 B cells, 22 cDCs, 13 maturation stages of T cells, 8 monocytes, 21 myeloid cells, 22 TBNKs, and 27 Tregs). Across 4 skin cancer classification phenotypes, 32 immune cells were associated with OMNS (15 negative and 17 positive correlations), 44 immune cells with MM (19 negative and 25 positive correlations), 36 immune cells with BCC (18 negative and 18 positive correlations), 24 immune cells with SCC (14 negative and 10 positive correlations).

Concerning the relationship between inflammatory cytokines and skin cancers, 17 significant associations were disclosed (P_IVW_ < 0.05), including 5, 2, 6, and 4 associations with OMNS, MM, BCC, and SCC, respectively. Among them, levels of causal cytokines C-C motif chemokine 4, TNF-beta, and TNF-related activation-induced cytokine were negatively connected to the risk of both OMNS and BCC, while cytokine Cystatin D was positively connected with the risk of OMNS and BCC.

Additionally, the results of the IVW method indicated that 17 antibody immune responses and 30 circulating metabolomics were causally associated with skin cancers, respectively. Specifically, 3, 4, 4, and 6 antibody immune responses were associated with OMNS, MM, BCC, and SCC, respectively, and 3, 2, 5, and 20 metabolomics exhibited a causal relationship with OMNS, MM, BCC, and SCC, respectively. The findings demonstrated 2 shared causal metabolites and 1 shared causal antibody for OMNS, BCC, and SCC: the level of Epstein-Barr virus ZEBRA antibody and ratio of 18:2 linoleic acid to total fatty acids were negatively related to these 3 skin cancer subtypes, and Merkel cell polyomavirus VP1 antibody level is positively related to them. Sensitivity analysis confirmed the robustness of all MR findings, showing no significant heterogeneity or horizontal pleiotropy (see Table S6, Supplemental Digital Content, https://links.lww.com/MD/P431, which illustrates heterogeneity and pleiotropy analyses of immune cells, inflammatory cytokines, circulating metabolites, and antibodies immune responses on skin cancers).

### 3.3. Mediation analysis results

To explore the causal pathway from blood cells to skin cancers mediated by immune cells, inflammatory cytokines, circulating metabolites, and antibodies immune responses, the identified cells, cytokines, metabolites, and antibodies associated with skin cancers from previously 2-sample MR analyses were applied for further explorations.

Firstly, the causal relationship among blood cells, immune cells, cytokines, metabolites, and antibodies was evaluated. We identified 28 associations of blood cells to immune cells (OMNS, 10; MM, 5; BCC, 12; SCC, 1), 4 associations to inflammatory cytokines (OMNS, 1; BCC, 2; SCC, 1), 3 associations to circulating metabolites (OMNS, 1; BCC, 2), 3 associations to antibodis (OMNS, 1; BCC, 2). The absence of horizontal pleiotropy and heterogeneity was validated for these MR results via the sensitivity analysis. Subsequently, multivariable MR analysis examined these mediators for their mediating effects, calculating indirect effects and proportions.

Excluding inconsistent direction of direct, indirect, and total effects, we finally identified 19 potential evidence (Table 2). Interestingly, it was discovered that WNR-chloroform 8h-PLA-FSS had similar modulation effects on the risk of OMNS and BCC through mediating CD8br NKT lymphocyte (7.9% proportion on OMNS and 6.4% proportion on BCC) and HLA DR on DC (8.1% proportion on OMNS and 6.7% proportion on BCC). Meanwhile, the risk of OMNS and BCC can be simultaneously regulated by WNR-Nigericin-WBC2-SSC and WDF-Alhydrogel-NE4-SFS through the levels of human herpes virus 7 U14 antibody and cytokine TNF-beta, respectively.

**Table 2:** Mediation effect of blood cells on skin cancers via immune cells, inflammatory cytokines, circulating metabolites, and antibodies immune responses.

Exposure	Mediator	Outcome	Total effect	Direct effect	Mediation effect (95% CI)	Mediation proportion	*P* value
WDF colchicine Eosinophil FSM	CCR2 on CD14 + CD16- monocyte	OMNS	−0.053	−0.052	−0.001 (−0.012, 0.009)	3%	.78
WNR chloroform 8h Platelet FSS	CD8br NKT %T cell	OMNS	−0.014	−0.013	−0.001 (−0.002, 0.000)	6%	.18
WNR chloroform 8h Platelet FSS	CD8br NKT %lymphocyte	OMNS	−0.014	−0.013	−0.001 (−0.003, 0.000)	8%	.18
WNR chloroform 8h Platelet FSS	HLA DR on plasmacytoid DC	OMNS	−0.014	−0.013	−0.001 (−0.002, 0.000)	5%	.13
WNR chloroform 8h Platelet FSS	HLA DR on DC	OMNS	−0.014	−0.012	−0.001 (−0.003, 0.000)	8%	.14
WDF alhydrogel Neutrophil 4 SFS	TNF-beta levels	OMNS	−0.051	−0.050	−0.001 (−0.003, 0.000)	3%	.15
WNR nigericin WBC SSC	Human herpes virus 7 U14 antibody levels	OMNS	0.041	0.034	0.007 (−0.007, 0.020)	17%	.34
RET ciproflaxin Reticulocyte SSM	IgD + CD38dim %B cell	MM	−0.085	−0.076	−0.009 (−0.019, 0.002)	10%	.10
RET ciproflaxin Reticulocyte SSM	CD4 + CD8dim AC	MM	−0.085	−0.080	−0.005 (−0.012, 0.002)	6%	.16
WDF KCI Neutrophil 2/Neutrophil 4 ratio	HLA DR + CD4 + AC	MM	−0.068	−0.067	−0.001 (−0.006, 0.003)	2%	.57
WDF colchicine Eosinophil FSM	BAFF-R on IgD+	BCC	−0.065	−0.063	−0.002 (−0.021, 0.017)	3%	.86
WNR chloroform 8h Platelet SFC	BAFF-R on B cell	BCC	−0.011	−0.011	−0.001 (−0.001, 0.000)	5%	.18
WNR chloroform 8h Platelet SFC	CD11b on CD14 + monocyte	BCC	−0.011	−0.010	−0.001 (−0.002, 0.000)	9%	.13
WNR chloroform 8h Platelet FSS	CD28- CD8br AC	BCC	−0.014	−0.013	−0.001 (−0.002, 0.000)	6%	.17
WNR chloroform 8h Platelet FSS	CD11b on CD14 + monocyte	BCC	−0.014	−0.013	−0.001 (−0.002, 0.000)	7%	.16
PLT-F baseline WBC SCM	TNF-beta levels	BCC	−0.049	−0.047	−0.001 (−0.003, 0.000)	3%	.12
WDF alhydrogel Neutrophil 4 SFS	TNF-beta levels	BCC	−0.042	−0.040	−0.002 (−0.003, 0.000)	4%	.12
WDF alhydrogel Neutrophil 4 SFS	Cytomegalovirus pp28 antibody levels	BCC	−0.042	−0.037	−0.005 (−0.011, 0.002)	11%	.14
WNR nigericin WBC SSC	Human herpes virus 7 U14 antibody levels	BCC	0.038	0.033	0.006 (−0.005, 0.017)	15%	.31

BCC = basal cell carcinoma, FSS = forward scatter standard, MM = malignant melanoma, OMNS = other malignant neoplasms of skin, SFC = side fluorescence coefficient, SFM = side fluorescence median, SFS = side fluorescence standard, SSC = side scatter coefficient, SSM = side scatter median, SSS = side scatter standard.

## 4. Discussion

Skin cancers have been associated with immune cells, inflammatory cytokines, metabolites, and antibodies in recent years; however, most findings are derived from case–control studies or experimental animal research, demonstrating correlations with skin cancers but failing to establish causal relationships. Using MR analysis, this study observed 18 human blood cells, 136 immune cells, 17 inflammatory cytokines, 17 antibodies, and 30 metabolites potentially causally linked to skin cancers (including OMNS, MM, BCC, and SCC). Conversely, reverse MR analysis indicated that skin cancers also significantly influenced the 4 above-identified blood cells. Mediation analysis further identified 14 immune cells, 3 inflammatory cytokines, and 3 antibodies involved in regulating blood cells in OMNS, MM, and BCC. This study represents the first comprehensive MR analysis investigating the causal relationships and mediation roles of blood cells, immune cells, inflammatory cytokines, metabolites, and antibodies in skin cancers.

According to previous reports, the progression of various cancers is significantly associated with the infiltration of blood cells.^[27,28]^ Meanwhile, these cells may play different roles in different tumors. For example, as one of the most common leukocytes in blood, neutrophils can promote the metastasis and growth of hepatocellular carcinoma through forming a tumor microenvironment and an inflammatory microenvironment,^[29]^ while they can also be cytotoxic to cancer cells by driving reactive oxygen species.^[30,31]^ These contradictory conclusions may result from confounding factors and reverse causal relationships. Thus, it is necessary to use big data analysis to clarify the relationship between blood cells and tumors, including skin cancers. Our findings suggest 18 association pairs between blood cells and skin cancers. Interestingly, it was discovered that the levels of neutrophils under alhydrogel perturbation and skin cancers, OMNS, and BCC were causally associated with each other. Alhydrogel neutrophils can significantly reduce the risk of OMNS and BCC, while the progression of OMNS and BCC can increase the level of alhydrogel neutrophils. This may be due to the characteristic that neutrophils will undergo a “change” in immunogenicity from anti-tumorigenic to pro-tumorigenic with the development of cancer.^[32]^ In early-stage melanomas, neutrophils will cross-present to CD8 + T cells to induce tumor-specific effector T cell responses, and eventually stimulate anti-tumor effects.^[33]^ However, over time, the ability of neutrophils to participate in immune pathways decreases with cancer progression.^[34]^ Thus, the specific role and mechanism of neutrophils in different stages of skin cancer need further exploration.

Apart from blood cells, this study also indicated several causal relationships between immune cells, inflammatory cytokines, antibodies, metabolites, and skin cancers. As critical hallmarks of malignancies' occurrence and development, multiple prior studies have indicated that tumor inflammation and immune evasion play a pivotal role in stages of cancer progression, including initiation, metastasis, invasion, and malignant conversion,^[6,35]^ and various immune and inflammatory cells and cytokines participate in these processes.^[36]^ Melanoma has been regarded as an immunogenic cancer for decades. When melanoma occurs, antigen-presenting cells, the primary mediators of skin immune surveillance, will present antigens to cytotoxic CD8^+^ T cells through HLA I. Thereafter, cytotoxic T cell responses can be activated by co-stimulatory molecules, such as CD28 on T cells.^[37,38]^ Recently, studies showed that interactions between immunity and melanoma are often tipped in favor of cancer development.^[39]^ Melanoma-associated immunosuppressive components, like myeloid-derived suppressor cells, alternatively activated macrophages (M2d), and Tregs, along with inflammatory cytokines such as TGF-β and IL-10, can divert antibody expression and B cell response in favor of suppressive subclasses like IgG4 and inhibit the anti-tumoral functions of macrophages, DCs, and cytotoxic T cells.^[40–42]^ Moreover, clinical investigations have discovered that the abnormal levels of numerous serum inflammatory cytokines, such as IFNα, IL family, and interferon-γ, are strongly connected to skin cancer metastasis, survival prognosis, and postoperative recurrence.^[39,43]^ Nevertheless, as inflammatory mechanisms are usually intertwined with autoimmune diseases, pathogenic infections, or other pathological conditions,^[44]^ it is difficult to clarify their independent effects on carcinogenesis and progression. Exploring the tumor specificity of the immune and inflammatory microenvironment cytokine in skin cancer might provide new insights for the treatment of malignant skin cancer patients. In this study, we identified 136 immune cells, 17 inflammatory cytokines, 17 antibodies, and 30 circulating metabolites that play a causal role in the risk of skin cancer. Among them, 13 immune cells, 3 inflammatory cytokines, and 3 antibodies also showed mediating effects from blood cells to skin cancers. Importantly, using the immune cell, antibody, inflammatory cytokine, and circulating metabolite levels as mediation factors in our study provides more comprehensive exploration of the role of the kinds of environment in skin cancers, thereby enhancing the study’s credibility.

This is the first study to comprehensively analyze the causal relationship among blood cells, immune cells, antibodies, inflammatory cytokines, circulating metabolites, and skin cancer subtypes. Additionally, a mediation pathway from blood cells to skin cancer was constructed through the other 4 factors. However, there are still several limitations. Firstly, the lack of individual information, such as sex and age, hinders further stratified analyses. Although we have pinpointed several blood cells, inflammatory cytokines, antibodies, circulating metabolome, and immune cells associated with the risk of skin cancers, the precise molecular mechanisms underlying these correlations remain unclear, and future analyses should account for such potential pathways to provide clearer insights. Secondly, the dataset only includes individuals of single ancestry, thus limiting racial heterogeneity in this study. Although the study is robust, it may not fully represent a wider population due to low participation rates. In future research, we need to expand the dataset to include a more diverse population range, thereby improving the generalizability and applicability of the findings to a wider population. Thirdly, it has to be acknowledged that the results of our study are primarily based on statistical analysis, which underscores that additional experimental and clinical analyses are necessary to validate our study findings.

## 5. Conclusion

In conclusion, this study supports the potential causal relationship between blood cells, immune cells, antibodies, inflammatory cytokines, circulating metabolites, and skin cancers through bidirectional MR analysis. Although its underlying mechanisms for the detected correlation need to be further elucidated, the findings still provide novel insights into skin cancer progression.

## Author contributions

**Conceptualization:** Jiahui Yao.

**Data curation:** Jiahui Yao.

**Formal analysis:** Jiahui Yao.

**Methodology:** Jiahui Yao, Mingshuai Han.

**Project administration:** Jiahui Yao.

**Validation:** Mingshuai Han.

**Visualization:** Mingshuai Han.

**Writing – original draft:** Mingshuai Han.

**Writing – review & editing:** Jiahui Yao.
